# Unravelling the relationship between the tsetse fly and its obligate symbiont *Wigglesworthia*: transcriptomic and metabolomic landscapes reveal highly integrated physiological networks

**DOI:** 10.1098/rspb.2017.0360

**Published:** 2017-06-28

**Authors:** XiaoLi Bing, Geoffrey M. Attardo, Aurelien Vigneron, Emre Aksoy, Francesca Scolari, Anna Malacrida, Brian L. Weiss, Serap Aksoy

**Affiliations:** 1 Department of Epidemiology of Microbial Diseases, Yale School of Public Health, New Haven, CT 06520, USA; 2 Department of Biology and Biotechnology, University of Pavia, 27100 Pavia, Italy

**Keywords:** tsetse, *Wigglesworthia* symbiosis, transcriptomic profiling, metabolomic analysis, vitamin biosynthesis, mutualism

## Abstract

Insects with restricted diets rely on obligate microbes to fulfil nutritional requirements essential for biological function. Tsetse flies, vectors of African trypanosome parasites, feed exclusively on vertebrate blood and harbour the obligate endosymbiont *Wigglesworthia glossinidia.* Without *Wigglesworthia*, tsetse are unable to reproduce. These symbionts are sheltered within specialized cells (bacteriocytes) that form the midgut-associated bacteriome organ. To decipher the core functions of this symbiosis essential for tsetse's survival, we performed dual-RNA-seq analysis of the bacteriome, coupled with metabolomic analysis of bacteriome and haemolymph collected from normal and symbiont-cured (sterile) females. Bacteriocytes produce immune regulatory peptidoglycan recognition protein (*pgrp-lb*) that protects *Wigglesworthia*, and a multivitamin transporter (*smvt*) that can aid in nutrient dissemination. *Wigglesworthia* overexpress a molecular chaperone (GroEL) to augment their translational/transport machinery and biosynthesize an abundance of B vitamins (specifically B_1_-, B_2_-, B_3_- and B_6_-associated metabolites) to supplement the host's nutritionally deficient diet. The absence of *Wigglesworthia's* contributions disrupts multiple metabolic pathways impacting carbohydrate and amino acid metabolism. These disruptions affect the dependent downstream processes of nucleotide biosynthesis and metabolism and biosynthesis of *S*-adenosyl methionine (SAM), an essential cofactor. This holistic fundamental knowledge of the symbiotic dialogue highlights new biological targets for the development of innovative vector control methods.

## 1. Introduction

The nature of symbiotic associations between microorganisms and animals range from mutualistic to parasitic interactions [1]. Many insects that subsist on diets with limited nutrients have evolved relationships with obligate symbionts that provide essential nutrients lacking in their diet [2]. Aphids and tsetse flies, two well-studied systems, feed exclusively on plant sap and vertebrate blood, respectively. The symbiotic association in both insects was established over 50 Ma [3], and, in both cases, led to unique cellular and biochemical adaptations that benefit both partners. Pea aphids rely on *Buchnera aphidicola* for essential amino acids low in plant phloem [4]*,* while tsetse obtain specifific blood-deficient nutrients (such as B vitamins) from *Wigglesworthia glossinidia* [5,6]. The functional contributions of each symbiosis are reflected in the modern-day *Buchnera* and *Wigglesworthia* genomes. Both endosymbionts are relatives of *Escherichia coli* in the γ-protebacteria class and their genomes have undergone dramatic erosion because of the initial association with their host insects. Despite this genomic reduction, the small genome of *Buchnera* (about 445 kb in size) has retained the ability to synthesize all essential amino acids [7,8], while the genome of *Wigglesworthia* (about 700 kb in size) encodes the enzymes of pathways involved in the synthesis of many B vitamins [9,10]. In exchange, the intracellular symbionts are protected from hostile gut immune responses and are provided with a steady supply of nutrients and an efficient vertical transmission route to progeny.

A holistic view of the molecular and biochemical dialogue between the two partners is lacking. Hence, we investigated this dialogue between *Wigglesworthia* and its partner, the tsetse fly. Tsetse females are viviparous, which is defined as reproduction via obligate intrauterine embryogenesis and larvigenesis, as well as the production and provisioning of all larval nutrition in the form of milk secretions by milk glands (differentiated accessory glands) [11]. *Wigglesworthia* lie free in the cytosol of specialized epithelial cells in the midgut (bacteriocytes), which collectively form the bacteriome organ [12]. To prevent the induction of antibacterial immune cascades that can damage *Wigglesworthia*, bacteriocytes produce high levels of the immune protein peptidoglycan recognition protein (PGRP-LB), which degrades the immune eliciting peptidoglycan [13]. In contrast to the bacteriome organ where the symbiont is intracellular, *Wigglesworthia* are extracellular in the lumen of the female's milk gland [14,15], and are transmitted during pregnancy to the intrauterine larva via the milk. Differential expression of *Wigglesworthia* genes have been noted from the bacteriome organ and the female milk, such as those that encode flagella-associated proteins which are produced only by extracellular *Wigglesworthia* [9], and may facilitate symbiont transmission to the larva [9,15].

*Wigglesworthia* are essential for the proper functioning of multiple host physiologies. The presence of the endosymbionts during juvenile development enhances tsetse's immune system maturation [16,17]. In the absence of *Wigglesworthia*, emerging adults are deficient in cellular immune responses and are more susceptible to trypanosome infections [18,19]. Besides immune enhancement, *Wigglesworthia* is required for tsetse's fecundity, as without this bacterium females are unable to support the development of their intrauterine larva. Loss of fecundity in the absence of *Wigglesworthia* can be rescued by dietary supplementation with yeast extract or to a lesser extent by supplementation with B vitamins [19]. One B vitamin, vitamin B6, is an essential cofactor for the enzyme alanine-glyoxylate aminotransferase (AGAT), which is required for biosynthesis of proline—the major soluble energy source in tsetse [20].

Here, we use transcriptomic and metabolomic analyses to develop a holistic understanding of the symbiotic dialogue that supports the fitness of the partnership at the molecular and biochemical levels. We discuss the core functions of the obligate symbiosis and the host physiological pathways dependent on this relationship to maintain the optimal host and symbiont homeostasis.

## 2. Results and discussion

We used a ‘dual RNA-seq’ approach to identify gene products putatively produced by both *Wigglesworthia* and tsetse bacteriocytes. Analysis of prokaryotic transcriptomes (bacteria and archaea) constitute a technical challenge because microbial mRNAs lack the 3′ poly(A) tail that is used to enrich eukaryotic mRNA. As rRNAs account for over 80% of cellular RNA, sequencing of total RNA without mRNA enrichment yields mostly non-mRNA sequences [21,22]. This issue is exacerbated when measuring gene expression of obligate intracellular symbionts residing in host tissues, and typically yields a low proportion of bacterial RNAs. Here, we enriched for both prokaryotic and eukaryotic mRNAs using an rRNA subtraction method to capture both host and symbiont transcripts from dissected intact bacteriome organs of *Glossina morsitans morsitans* (hereafter called *Glossina*).

### (a) Host–symbiont dialogue promotes symbiont well-being and nutritional homeostasis

We obtained 22 million high-quality reads from each of the three biological bacteriome replicates. On average 36% of the reads mapped to the *Wigglesworthia* genome and 17% to the predicted *Glossina* transcriptome (GmorY1.4 obtained from VectorBase) (electronic supplementary material, figure S1 and table S2). A significant proportion of reads (16%) mapped to unannotated regions of the *Glossina* genome suggesting expression of unannotated genes. The de novo assembly of the remaining unmapped reads into contigs revealed that some of these reads (1.34%) are homologous to viral genes (electronic supplementary material, table S3). The majority of the reads mapping to the *Wigglesworthia* genome (approx. 88%) and *Glossina* transcriptome (approx. 98%) were associated with mRNA coding sequences (CDSs), which confirms the efficiency of the rRNA elimination process.

We identified bacteriocyte-enriched transcripts by comparing bacteriome transcriptomes to whole midgut transcriptomes [23], and then mined this dataset to unravel bacteriocyte-associated adaptations and symbiont contributions to host physiology. Principal component analysis of the expression data shows clear differentiation between the bacteriome and gut samples with little variance between the replicates (electronic supplementary material, figure S2). To identify bacteriocyte-enriched genes, we used the LOX (Level Of eXpression) software package to compare bacteriome and midgut transcriptome data. LOX uses a Markov chain Monte Carlo algorithm to calculate and compare gene expression levels and can compare for differential/enriched expression between transcriptomes from different tissues or between those that use different sequencing methods [24]. We identified 252 *Glossina* transcripts that are enriched in bacteriocytes (LOX *p*-value < 0.025 and greater than fivefold RPKM ratio in Bac/Gut comparison) and determined the putative molecular functions of these gene products by gene ontology (GO) analysis (electronic supplementary material, figure S3 and table S4).

The most abundant bacteriocyte-enriched products are associated with the ion binding, molecular function and transmembrane transporter activity categories. These include a putative lectin (*salivary C-type lectin GMOY000466*), two sodium/potassium pumps (GMOY004651, GMOY003579), a metalloprotease (GMOY009531), two putative proteins related to vesicular transport/exocytosis (a homologue of an exosome component: GMOY011640 [25] and Ras signalling pathway (14-3-3 zeta, isoform D - GMOY006173)) and the immune regulatory PGRP-LB (*GMOY006730*).

Lectins are carbohydrate-binding proteins that can recognize bacterial surface glycoproteins to facilitate symbiont specificity, localization and recognition [1]. In the marine nematode *Laxus oneistus*, lectins are predicted to mediate the specific recruitment of its symbionts [26]. In the octocoral *Sinularia lochmodes,* lectins play a role in the acquisition of an endosymbiotic dinoflagellate and its subsequent transformation into a non-motile symbiotic form [27]. In addition, recent work in mosquitoes shows lectins are associated with the colonization and protection of gut microbiota [28].

Abundant expression of PGRP-LB in tsetse's bacteriome was previously noted, and functional experiments confirmed the protein's role as an immune modulator, a symbiont safeguard [13,29] and a trypanocidal effector molecule that enhances tsetse's resistance to fitness reducing parasite infections [29]. A similar immunoregulatory role for PGRP-LB was described in symbioses associated with the weevil *Sitophilus zeamais* [30] and the squid *Vibrio fischeri* [31]. The role of PGRPs in mediating host tolerance to symbiosis, inflammation and immune system maturation is now being recognized in multiple eukaryotic systems [32].

Transmembrane proteins are another dominant category of bacteriocyte-enriched gene products, and include amino acid transporters (*proton-coupled amino acid transporter, excitatory amino acid transporter 1*), Na^+^ /K^+^ pumps, a transient receptor potential cation channel (*trpm*) as well as the sodium-dependent multivitamin transporter (*smvt* or *Slc5a8*) and sodium-coupled solute transporter 1 (electronic supplementary material, figure S3 and table S4). In the pea aphid, bacteriocytes also preferentially express mitochondrial transporters, amino acid transporters and Rab, a protein that regulates vesicular transport [33,34]. In the weevil bacteriocytes, transcripts essential for trafficking gene products, such as SNARE and Rab, have also been noted [35]. Thus, the bacteriocytes appear to enhance the symbiosis by sheltering *Wigglesworthia* and ensuring that symbiont-produced compounds are effectively mobilized for utilization by other host tissues.

**Figure 1.**
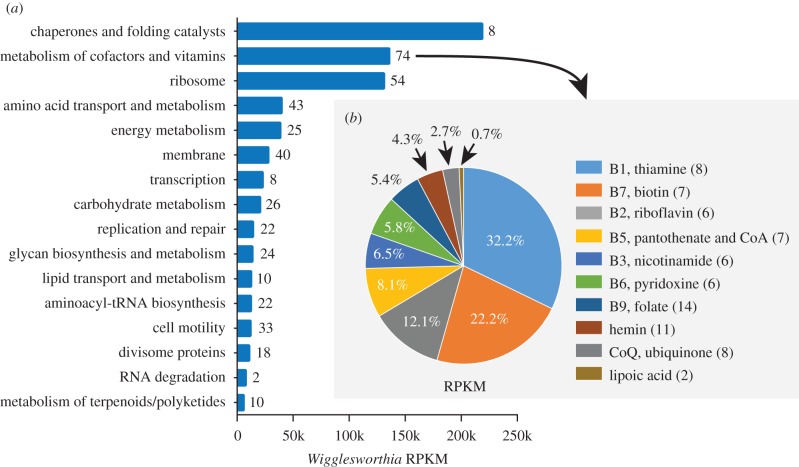
KEGG analysis of *Wigglesworthia* CDS genes. (*a*) Bars represent the sum of gene RPKM values associated with respective KEGG categories. Numbers represent the number of genes per category. (*b*) Proportion of the gene expression associated with *Wigglesworthia* cofactors and vitamin biosynthesis. The number of genes per corresponding pathway is shown in parenthesis. Bars represent the sum of the RPKM values of the genes (see also the electronic supplementary material, table S5).

Long-term symbiosis poses a number of challenges for symbiotic fitness, such as the population bottlenecks experienced during the process of vertical transmission to progeny, and the functional erosion resulting from genomic reductions common in obligate symbionts. As noted previously in the tsetse/*Wigglesworthia* [36,37], aphid/*Buchnera* [38] and weevil/*Sodalis* [39] symbioses, transcripts encoding GroEL/GroES (associated with ‘chaperone and folding catalysts’) are the most abundant in the bacteriome data (figure 1*a*; electronic supplementary material, table S5). Beyond *chaperonins,* almost 10% of *Wigglesworthia* reads matched to a non-coding transfer–messenger RNA (tmRNA, or *ssrA*) and its unique binding protein (*smpB*) (electronic supplementary material, table S2), which are involved in the degradation of non-functional peptides [40]. Thus, the high expression of chaperons and tmRNP can alleviate the negative effects accumulating nucleotide substitutions can have on protein function(s) in the absence of rigorous DNA repair systems retained in the functionally eroded obligate symbiont genomes [41]. The next most abundant category in the *Wigglesworthia* transcriptome encodes functions associated with ‘metabolism of cofactors and vitamins’, the majority of which are associated with vitamin B-related products, including vitamin B7 biotin (*bioB* and *bioF*), B1 thiamine (*thiC*, *thiE, thiF* and *thiG*), B2 riboflavin (*ribB* and *ribC*) and B5 pantothenate (*panB*) (figure 1*b*; electronic supplementary material, table S5). Two other abundant nutrient biosynthesis-related transcripts were *metK,* essential for methionine–cysteine metabolism, and *acpP*, involved in fatty acid biosynthesis (electronic supplementary material, table S5). Thus, the transcriptome data validate previous physiological studies, which predicted a role for *Wigglesworthia* produced vitamin metabolites in host dietary supplementation and fecundity [9,20,42].

### (b) Bacteriocyte enriched genes are differentially expressed under aposymbiotic and trypanosome infected conditions

**Figure 2.**
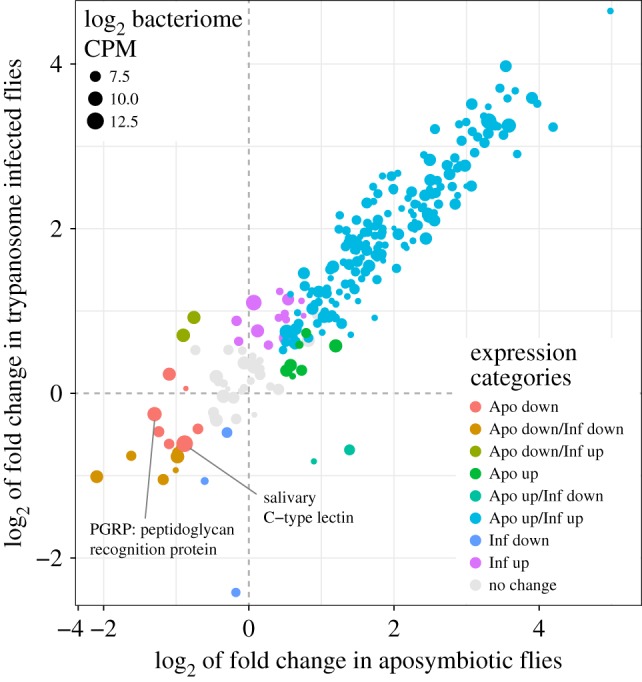
Differential expression of bacteriocyte-enriched genes in aposymbiotic- and trypanosome-infected flies. This graph represents the differential expression of the 252 bacteriocyte-enriched genes in the gut tissue of aposymbiotic flies (*x*-axis) and trypanosome-infected flies (*y*-axis). Data point size represents expression level (log_2_ of counts per million (CPM) values) in the bacteriome-specific transcriptome. Transcripts were categorized as up- or downregulated if scored as differentially expressed by edgeR analysis with a *p*-value of less than 0.05 and a false discovery rate (FDR) score of less than 0.05 (see also the electronic supplementary material, figure S4 and table S6 for detailed annotations).

To determine whether transcripts identified as bacteriocyte enriched are responsive to different physiological states, we analysed the expression profile of bacteriocyte enriched genes in gut tissues (including bacteriome) of aposymbiotic (adults that have undergone juvenile development in the absence of symbionts, termed aposymbiotic adults) and trypanosome-infected flies, as both states result in reduced host fecundity [19,43]. A positive correlation exists between *Wigglesworthia* density during tsetse development and expression of the fly's resistance to trypanosomes [29]. Additionally, aposymbiotic flies are highly susceptible to trypanosomes [18]. We noted that of the 252 bacteriocyte-enriched transcripts, 182 are upregulated in both aposymbiotic and parasitized conditions (electronic supplementary material, figure S4 and table S6). The similarity in the transcriptional response of these gene products could result from the host's response to the lack of specific nutrients in the absence of *Wigglesworthia,* or in competition for nutrients in the presence of trypanosomes. In fact, trypanosomes in the tsetse midgut rely on l-proline as their main carbon source [44], which is also used by the fly for lactation and to fuel flight muscles [20]. Our analysis of haemolymph from normal and infected flies showed a significant decrease in proline levels in parasitized individuals [20], indicative of this competitive nutritive interaction. Of interest, expression of *pgrp-lb* and the *salivary c-type lectin* were both downregulated in the absence of symbionts, but not in the presence of parasites (figure 2). The downregulated expression profile of these genes in the absence of *Wigglesworthia* could be due to the putative roles these gene products have in fostering and maintaining the symbiotic partnership in tsetse and other organisms [13].

### (c) *Wigglesworthia* provides vitamin B metabolites to its partner

**Figure 3.**
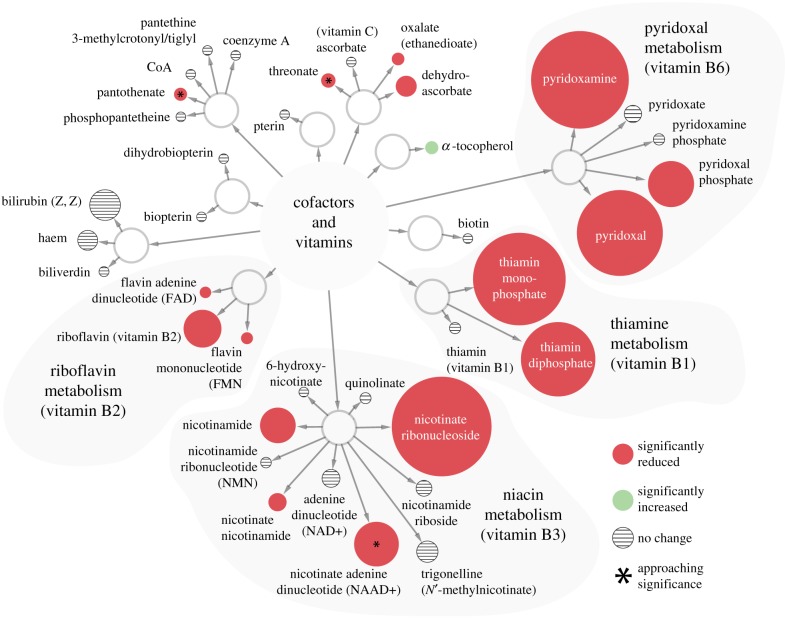
Differential cofactor and vitamin abundance in bacteriomes of symbiotic and symbiont-cured tsetse. Circles represent compounds and metabolites. Connections represent related compounds/metabolites. Size represents the relative difference in metabolite abundance between symbiotic and symbiont-cured bacteriomes. Significance was determined by Welch's two-sample *t*-test with a *p*-value of <0.05. Numerical representation of this data is in Table S7.

To understand the physiological roles of host and symbiont products, we performed a metabolomic analysis of bacteriomes and haemolymph collected from normal (hereafter called symbiotic) and tetracycline treated adults (hereafter called symbiont-cured flies; figure 3). The metabolomic analysis clearly validates that vitamin supplementation is an essential core function of the symbiosis. For this analysis, tissue samples were screened for the presence and quantity of metabolites by mass spectrometry using a panel of approximately 1000 compounds that included a broad array of metabolite classes such as amino acids, peptides, carbohydrates, lipids, nucleotides, cofactors/vitamins and xenobiotics. Relative differences between samples were determined by comparison of mean metabolite abundance across four biological replicates. The analysis detected 38 compounds designated as vitamins/cofactors and showed that B vitamins are abundant in symbiotic bacteriomes and significantly reduced in the symbiont-cured bacteriomes (electronic supplementary material, table S7) and haemolymph (electronic supplementary material, table S8). In particular, metabolites and final products associated with vitamins B1 (thiamine), B2 (riboflavin/FAD) and B6 (pyridoxal-phosphate) were reduced in symbiont-cured samples (13%, 25% and 20% of the symbiotic levels, respectively). The dramatically reduced levels of the vitamin products detected in the haemolymph of symbiont-cured flies indicate their provisioning by *Wigglesworthia* endosymbionts. The B-vitamin group includes a diverse array of compounds that function as essential cofactors for enzymes associated with multiple biochemical pathways. In particular, we saw dysfunction in the glycogen metabolism and the pentose phosphate pathways (PPP) as well as the nucleotide biosynthesis and cysteine/methionine metabolism pathways responsible for the biosynthesis of the cofactor *S*-adenyl-methionine (SAM), as we describe further.

### (d) Glycogen phosphorylase activity is impaired in the absence of *Wigglesworthia*

The glycogen pathway catabolizes glycogen to glucose-6-phosphate for use in downstream pathways (electronic supplementary material, figure S5). In symbiont-cured bacteriomes, we observed an overabundance of the metabolite maltopentaose and reduced levels of maltotetraose, maltotriose and maltose. These changes indicate a disruption in the flies' ability to catabolize glycogen-derived metabolites (electronic supplementary material, table S7). Both the glycolysis pathway and PPP are dependent on glycogen catabolism for proper function. Glycogen phosphorylase (EC:2.4.1.1, *GMOY007990*), which catalyses the rate limiting step in the conversion of glycogen to glucose-6-phosphate subunits, is dependent on pyridoxal phosphate (vitamin B6) for its enzymatic function. Interestingly, the expression of the gene for glycogen phosphorylase (EC:2.4.1.1, *GMOY007990*) was also reduced in aposymbiotic fly guts to about 60% of control levels (electronic supplementary material, table S9).

### (e) Phosphoribosyl pyrophosphate levels are impaired in the absence of *Wigglesworthia*

**Figure 4.**
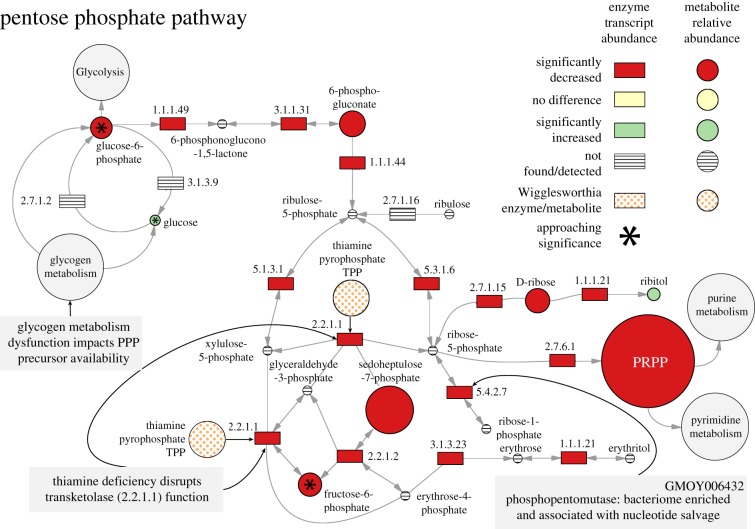
Differential metabolite abundance and enzyme gene expression in the pentose phosphate pathway between control and symbiont-cured tsetse. Rectangles represent enzymes and are defined by their KEGG enzyme ID numbers and circles represent metabolites/cofactors. Circle size represents the relative difference in abundance between symbiotic and symbiont-cured bacteriomes. Numerical representation of metabolite data is in Table S7 and enzyme gene expression data are in Table S9.

The PPP is a conserved multifunctional pathway that is central to cellular biosynthetic metabolism and in maintaining oxidative homeostasis [45] (figure 4). The primary input to this pathway, glucose-6-phosphate, is converted to ribulose-5-phosphate in a reaction that generates NADPH and helps to buffer oxidative stress. Ribulose-5-phosphate, in turn, functions as the primary substrate for the biosynthetic arm of PPP and is converted to ribose-5-phospate or erythrose-4-phosphate by the transketolase enzyme (EC: 2.2.1.1, GMOY007053) [46]. Transketolase is dependent upon thiamine as a cofactor, another of the B vitamins deficient in symbiont-cured flies. The activity of this enzyme directs the biosynthesis of precursors towards either nucleotide biosynthesis (ribose-5-phosphate to phosphoribosyl pyrophosphate (PRPP)) or aromatic amino acid biosynthesis (sedoheptulose-7-phosphate to erythrose-4-phosphate). In the symbiont-cured state, PRPP and sedoheptulose-7-phosphate are found at 0.62% and 18% of the amount detected in control flies, respectively (figure 4 and electronic supplementary material, table S7). Under aposymbiotic conditions, the expression of the majority of genes coding for enzymes associated with PPP metabolism are downregulated (electronic supplementary material, table S9). One of these enzymes, phosphomannomutase (EC: 5.4.2.7, *GMOY006432*), also associated with the purine metabolism pathway, is enriched in the bacteriocytes (electronic supplementary material, table S6). This enzyme converts ribose-1-phosphate derived from catabolized pyrimidine bases to ribose-5-phosphate which feeds into the production of PRPP [47]. Glycogen metabolism dysfunction disrupts the flow of glucose-6-phosphate molecules into the pathway and thiamine deficiency disrupts transketolase function. Thus, in symbiont-cured flies the PPP is negatively impacted in two aspects, the reduced availability of precursor molecules and enzyme functionality.

### (f) Nucleotide metabolism and salvage is impaired in the absence of *Wigglesworthia*

**Figure 5.**
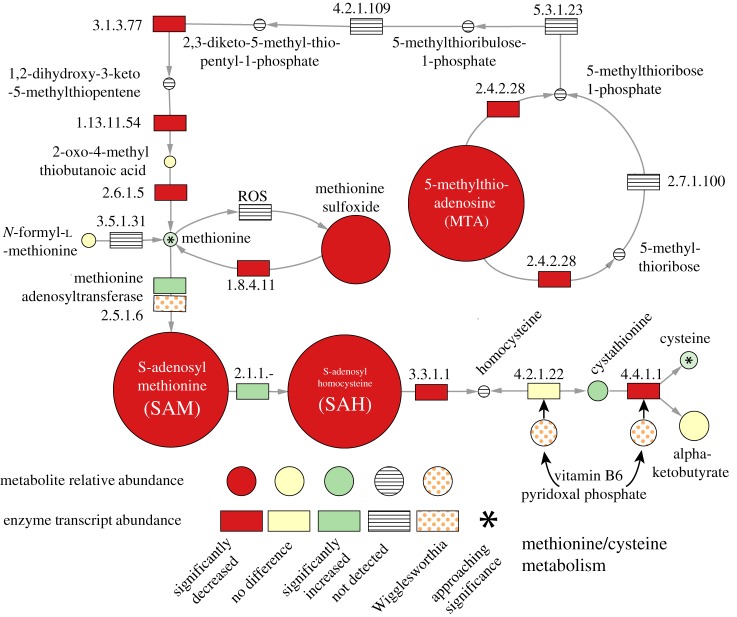
Differential metabolite abundances and enzyme associated gene expression in the methionine—cysteine metabolism pathway between control and symbiont-cured tsetse. Symbol descriptions are as described in figure 4. Numerical representation of metabolite data is in Table S7 and enzyme gene expression data are in Table S9.

The reduced levels of PRPP (0.62%) derived from the PPP in symbiont-cured tsetse impacts the function of both the purine and pyrimidine nucleotide metabolism pathways (electronic supplementary material, table S7a, figures S6 and S7). We noted substantial reductions in multiple metabolites associated with these pathways with the most dramatic decreases evident in the levels of critical products of the purine pathway: adenine (11%), adenosine (11%) and adenosine monophosphate (AMP) (28%) and the pyrimidine pathway: orotate (0.02%) and uridine monophosphate (12%). The observed reduction of AMP likely also results in reduced cyclic AMP (cAMP) levels. This has implications for the glycogen metabolic pathway as transcription of the gene for glycogen phosphorylase (EC:2.4.1.1, *GMOY007990*) is regulated by cAMP levels [48]. Gut gene expression associated with the enzymes in these pathways also differed from control flies as the majority were either up- or downregulated in relative transcript abundance in aposymbiotic flies (electronic supplementary material, table S9). Gene expression for one of these enzymes, adenine phosphoribosyltransferase (EC: 2.4.2.7, *GMOY001635*), is transcriptionally enriched in the bacteriocyte and is required for the salvage and recycling of degraded nucleotides by combining PRPP with adenine to produce AMP [49]. The fact that two of the bacteriocyte-enriched genes are associated with nucleotide salvage, *phosphomannomutase* (in the PPP) and *adenine phosphoribosyltransferase* (in the purine metabolism pathway), suggests that salvage and recycling of nucleotides may be an important function in the bacteriome. Transcript levels for both of these genes are downregulated in the aposymbiotic state (figure 5; electronic supplementary material, S4 and table S9). *Wigglesworthia's* genome encodes the enzymes required for both purine and pyrimidine biosynthesis suggesting that the bacterium may be assisting with nucleotide production [10]. A similar symbiotic relationship is observed between the deep-sea tube worm *Riftia pachyptila* and its bacterial endosymbiont. In this relationship, the bacteria produce pyrimidines de novo and intermediate metabolites for nucleotide salvage by host tissues throughout the worm [50]. The dramatic depletion of intermediate metabolites, such as PRPP and orotate, suggest that *Wigglesworthia* may act as a source of de novo nucleotide synthesis and producer of metabolic intermediates crucial for the function of enriched nucleotide salvage pathways.

### (g) Haemolymph amino acid levels are impaired in the absence of *Wigglesworthia*

We also analysed levels of free amino acids and associated metabolites in the haemolymph of symbiont-cured tsetse. This analysis showed an across the board decrease in these compounds (electronic supplementary material, table S8). Dysfunction in the PPP likely impacts the production of carbohydrate-derived precursor molecules required for amino acid biosynthesis, such as erythrose-4-phosphate. Although erythrose-4-phosphate was not detected in the metabolic panel, the level of its precursor sedoheptulose-7-phosphate in symbiont-cured bacteriome and haemolymph is only 20% of that of controls (electronic supplementary material, table S7 and S8). If erythrose-4-phosphate levels are similarly reduced, it would have negative implications for the biosynthesis of aromatic amino acids. We have previously shown that vitamin B6 deficiency impacts the critical alanine/proline shuttle system, which functions as the primary source of soluble energy in tsetse and is dependent on the enzyme AGAT, requiring vitamin B6 as a cofactor [20]. Vitamin B6 is also a cofactor for all other transaminase and decarboxylase enzymes involved in the metabolism of all amino acids [51]. These results collectively indicate a large-scale negative impact on the global metabolism of amino acids in the absence of critical vitamin metabolites.

### (h) Production of the co-factor *S*-adenosyl methionine is impaired in the absence of *Wigglesworthia*

**Figure 6.**
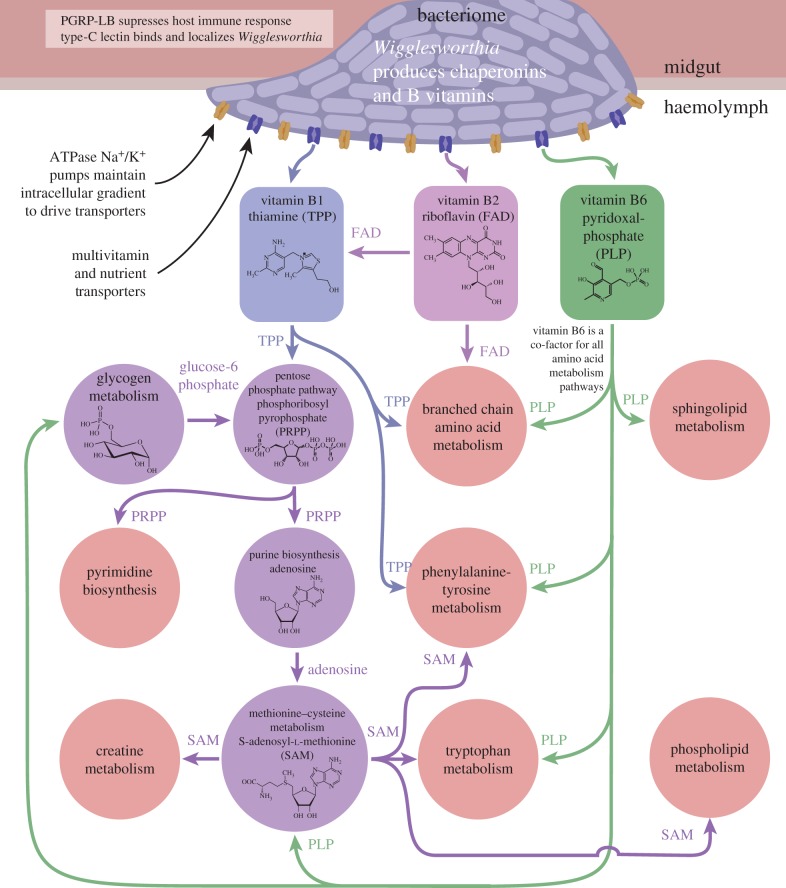
Schematic overview of the core symbiont functions and the host metabolic pathways dependent on *Wigglesworthia*-derived vitamin products. Arrows represent dependency of downstream pathways on vitamins (rectangles) or precursor molecules (circles) from the upstream process.

Downstream of the purine pathway, the methionine–cysteine metabolism pathway synthesizes the essential methylation cofactor *S*-adenosyl methionine (SAM; figure 5). The usage of SAM as a cofactor in metabolic reactions is second only to that of ATP [52]. The biosynthesis of SAM is performed by the methionine adenosyltransferase enzyme (EC 2.5.1.6, *GMOY012020*), which joins methionine and ATP to produce SAM. In aposymbiotic flies, gene activity associated with enzymes in this pathway are down with the exception of methionine adenosyltransferase, which is upregulated. In mammals, transcription of the gene coding methionine adenosyltransferase is under negative regulation by methionine [53]. Although methionine levels in symbiont-cured bacteriome samples are not significantly different from controls, methionine levels in the haemolymph are only about 25% of that of controls (electronic supplementary material, tables S7–S9). Of interest, one of the most highly expressed genes in the *Wigglesworthia* transcriptome (*metK*) also encodes methionine adenosyltransferase (electronic supplementary material, table S5), suggesting that *Wigglesworthia* may be assisting in the production of this cofactor. This pathway also involves two vitamin B6-dependent enzymes, cystathionine gamma-lyase (EC 4.4.1.1, *GMOY011763*) and cystathionine beta-synthase (EC 4.2.1.22 *GMOY005979*), that combine α-ketobutyrate and cysteine to generate cystathionine and then cystathionine to homocysteine. Homocysteine is then converted to methionine with the assistance of folate (another B vitamin not included in the panel of screened metabolites). In the symbiont-cured bacteriome, the combined effect(s) of vitamin B deficiencies on metabolism of amino acids and purines disrupt SAM biosynthesis. Levels of SAM and its demethylated form, *S*-adenosyl-l-homocysteine, in symbiont-cured bacteriomes are at 6% and 9% of that of control flies, respectively (figure 6 and electronic supplementary material, table S7). Given that methylation reactions are essential for amino acid biosynthesis, creatine and phospholipid metabolic pathways among others, the deficiency of this key cofactor results in negative implications for many downstream metabolic pathways in tsetse. The absence of *Wigglesworthia's* metabolic contributions results in an overall disruption of the fly's nutritional homeostasis. This disruption prevents the mother from providing the nutrients required to support the growth of intrauterine larvae.

## 3. Conclusion

The symbiosis between tsetse and *Wigglesworthia* is ancient and essential, as evidenced by the extensive adaptations each partner has developed to optimize and preserve this relationship (figure 6). The findings in this work expose previously unknown details of the metabolic coevolution the symbiosis represents and the integrated mutual dependence these organisms have for each other for survival. The absence of *Wigglesworthia*-derived B vitamins has a plethora of direct and indirect effects on *Glossina* metabolism, physiology and ultimately on the survival of the species. These effects highlight the dependencies of *Glossina's* physiology on processes, including nucleotide, amino acid and cofactor biosynthesis. These dependencies are potentially exploitable for the purpose of vector control through the targeted development of specific inhibitory compounds. Beyond vitamins, the genetic/physiological adaptations, such as reliance on chaperonins for protein synthesis in *Wigglesworthia,* or the enrichment of nutrient transport mechanisms by *Glossina's* bacteriocytes, also have the potential for exploitation. The relationship between *Glossina* and *Wigglesworthia* highlights an obligate dependence on the microbial partnership that results from reliance on a sole nutritional source, such as blood. The long-term coevolutionary association has led to a multitude of dynamic molecular and biochemical interactions that ensures the optimal fitness of the partnership.

## 4. Material and methods

### (a) Biological materials

*Glossina m. morsitans* wild-type (WT) flies, *Glossina* aposymbiotic flies (Apo) and symbiont-cured (tetracycline treated flies) were maintained as described in the electronic supplementary material, Materials and methods. Mammalian bloodstream form (BSF) parasites of *Trypanosoma brucei rhodesiense* (YTat 1.1) were expanded in rats as described [54].

### (b) Bacteriome transcriptomic analysis

Bacteriomes were dissected from females at around 20 days post-eclosion. Three biological replicates of 10 bacteriomes were collected. Dual RNA-seq libraries were prepared with ScriptSeq Complete Gold Kit (Epidemiology) (Epicentre, Madison, WI) and sequenced (75 bp single-end read) on Illumina HiSeq 2000 by Yale University Center of Genome Analysis (YCGA, New Haven, CT). Reads were mapped to *Wigglesworthia* genome (NC_016893) and to *Glossina* transcripts (version 1.4 obtained from Vectorbase [55], respectively, using CLC Genomics Workbench (CLC Bio, Cambridge, MA). We used the RPKM as a measure of relative gene expression [56]. Bacteriocyte-enriched *Glossina* genes were selected by comparing the ratio of RPKM expression values of transcripts between bacteriome-specific and previously published whole midgut transcriptomes [23]. *Glossina* transcripts were identified as enriched using the following parameters: bacteriome RPKM/whole gut RPKM ratio of greater than 5 and an average bacteriome RPKM of greater than 50. Statistical significance of gene enrichment was evaluated using the LOX software package [24]. Transcripts determined to be bacteriocyte enriched were annotated with GO terms using the Blast2GO software package [57]. *Wigglesworthia*-specific gene expression profiles were performed with KEGG pathway analysis [58]. Sequencing data are available in the Sequence Read Archive (SRR3956922-7) and detailed study protocols are described in the electronic supplementary material.

### (c) Comparative analysis of bacteriocyte-enriched gene expression in aposymbiotic- and trypanosome-infected guts

Whole tsetse gut transcriptomes (including cardia, bacteriome and midgut) were generated from control symbiotic females, aposymbiotic females and trypanosome infected females, respectively. Each treatment was represented by three replicate datasets. The sequence data for these transcriptomes are available in the Sequence Read Archive (aposymbiotic guts: SRR207250–SRR207252, trypanosome infected guts and controls: SRR3425153–SRR3425169). Detailed treatment and study protocols are described in the electronic supplementary material.

### (d) Metabolomic analysis

Tissues (bacteriome and haemolymph) were dissected from WT flies that received normal blood meals for five weeks and from WT flies that received four tetracycline supplemented blood meals (25 µg ml^–1^) post-eclosion followed by normal blood meals for four weeks (termed symbiont-cured). At the time of dissection all symbiont-cured flies had ceased larval deposition. Samples were shipped to Metabolon (Morrisville, NC) and analysis was performed using the DiscoveryHD4 global metabolomics platform. Detailed study protocols are described in the electronic supplementary material.

## Supplementary Material

Figure S1: Read-mapping statistics of bacteriome RNA-Seq Data

## Supplementary Material

Figure S2: PCA analysis of gut and bacteriome RNA-Seq Datasets

## Supplementary Material

Figure S3: Gene ontology analysis of bacteriocyte-enriched products associated with molecular functions

## Supplementary Material

Figure S4: Differential expression of bacteriocyte enriched genes in aposymbiotic and trypanosome infected flies with additional annotations

## Supplementary Material

Figure S5: Differential metabolite abundances and enzyme transcription between control and symbiont-cured tsetse in the glycogen metabolism pathway

## Supplementary Material

Figure S6: Differential metabolite abundances and enzyme associated gene expression in the purine metabolism pathway between control and aposymbiotic tsetse

## Supplementary Material

Figure S7: Differential metabolite abundances and enzyme associated gene expression in the pyrimidine metabolism pathway between control and aposymbiotic tsetse

## Supplementary Material

Table S1: Transcriptomic and metabolomic datasets utilized in this study

## Supplementary Material

Table S2: Mapping Statistics of bacteriome RNA-Seq Reads to *Wigglesworthia* and *Glossina* Genomes

## Supplementary Material

Table S3: BLAST analysis of viral sequences derived from *de novo* assembly from unmapped RNA-seq reads

## Supplementary Material

Table S4: Gene ontology analysis of *Glossina* genes with bacteriome enriched expression

## Supplementary Material

Table S5: KEGG functional analysis of *Wigglesworthia* genes

## Supplementary Material

Table S6: Annotation and differential expression of *Glossina* genes with bacteriome enriched expression

## Supplementary Material

Table S7: Bacteriome metabolome data

## Supplementary Material

Table S8: Hemolymph metabolome data

## Supplementary Material

Table S9: Differential expression of *Glossina* enzyme coding genes in control and aposymbiotic flies

## Data accessibility

Supporting figures and tables for this article including complete metabolomic datasets, GO analysis and annotation of bacteriome-enriched genes, and differential expression analysis results are included in the electronic supplementary material. RNA-seq datasets have been uploaded to the NCBI Sequence Read Archive (SRA) database. The SRA identification numbers for the datasets used in this article are as follows. Bacteriome RNA-seq: SRR3956925-7, aposymbiotic whole gut RNA-seq: SRR207250-2, trypanosome-infected whole gut RNA-seq: SRR3425153- SRR3425169.
