# Supplementary figures and images for: Unravelling the relationship between the tsetse fly and its obligate symbiont Wigglesworthia: transcriptomic and metabolomic landscapes reveal highly integrated physiological networks
Source: Proc Biol Sci. 2017 Jun 28;284(1857):20170360. doi: 10.1098/rspb.2017.0360 (PMC5489720; doi:10.1098/rspb.2017.0360)

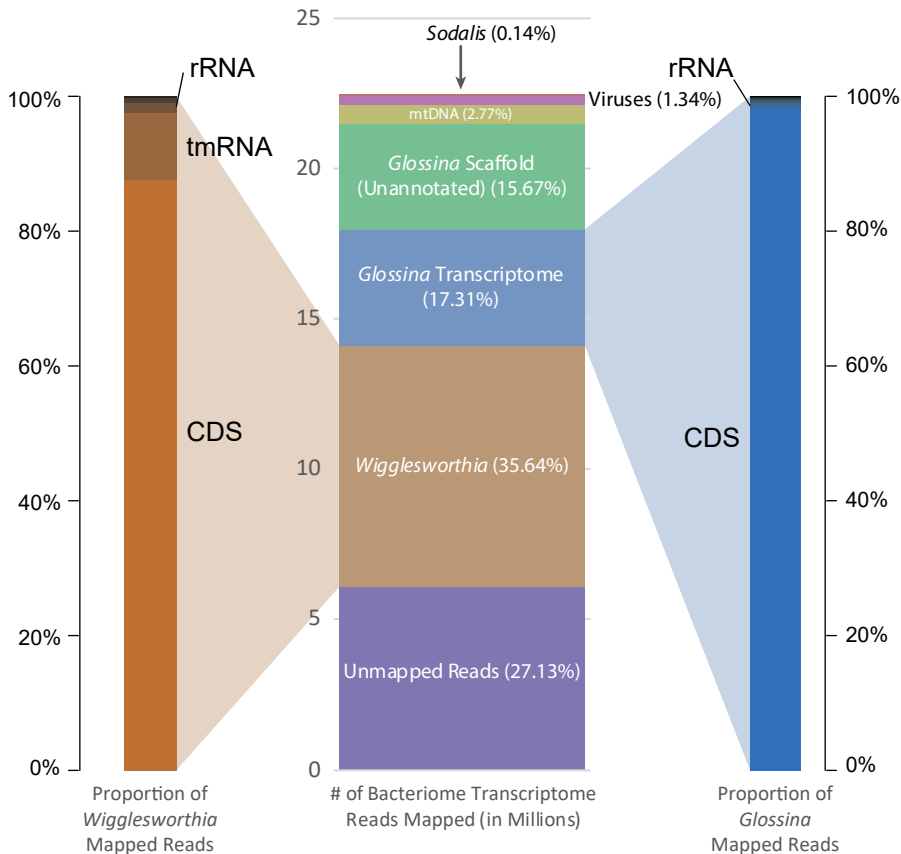

Supplement: Figure S1: Read-mapping statistics of bacteriome RNA-Seq Data [file rspb20170360supp1.pdf]

Principal Component Analysis of Whole Gut Transcriptomes vs Bacteriome Transcriptomes

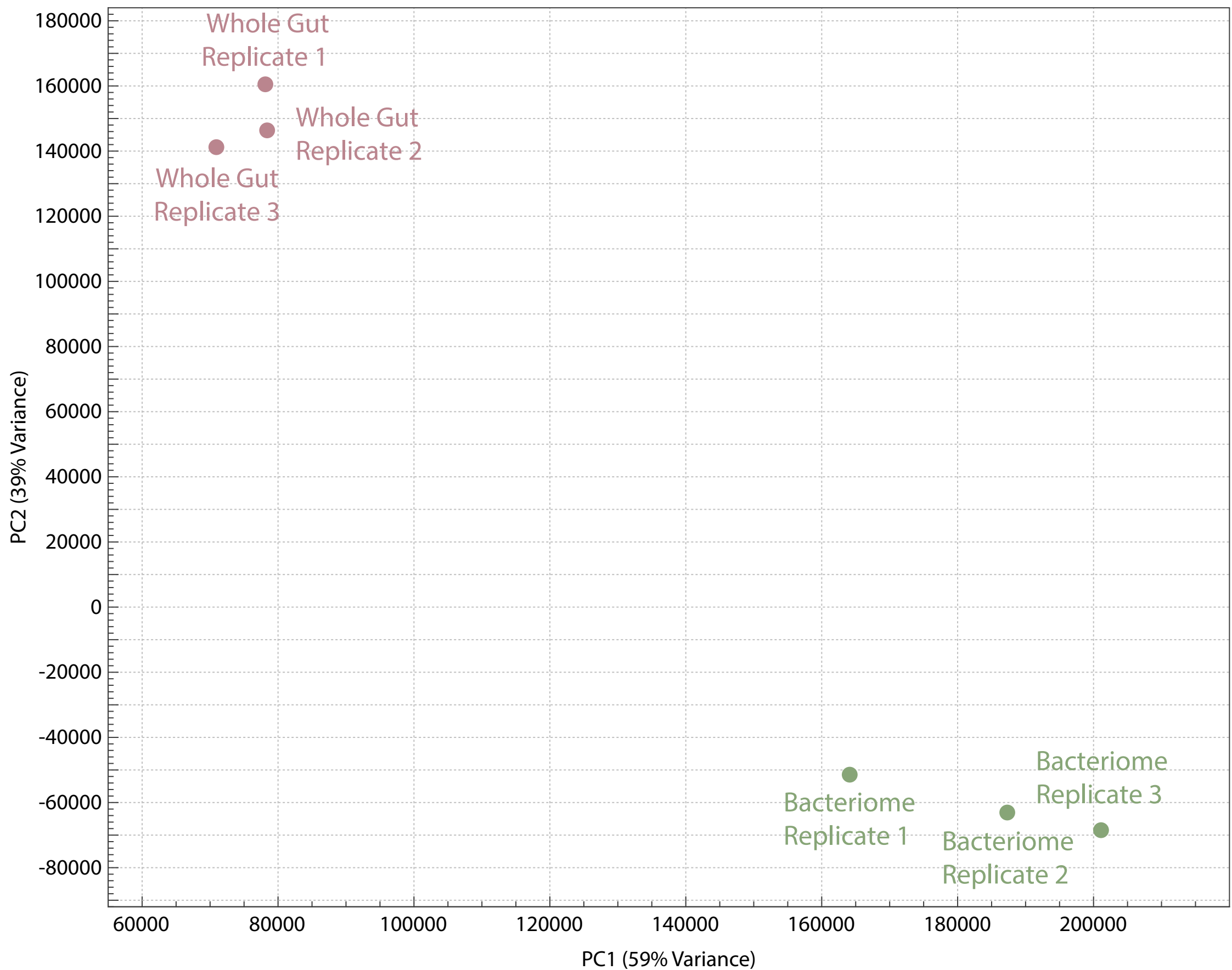

Supplement: Figure S2: PCA analysis of gut and bacteriome RNA-Seq Datasets [file rspb20170360supp2.pdf]

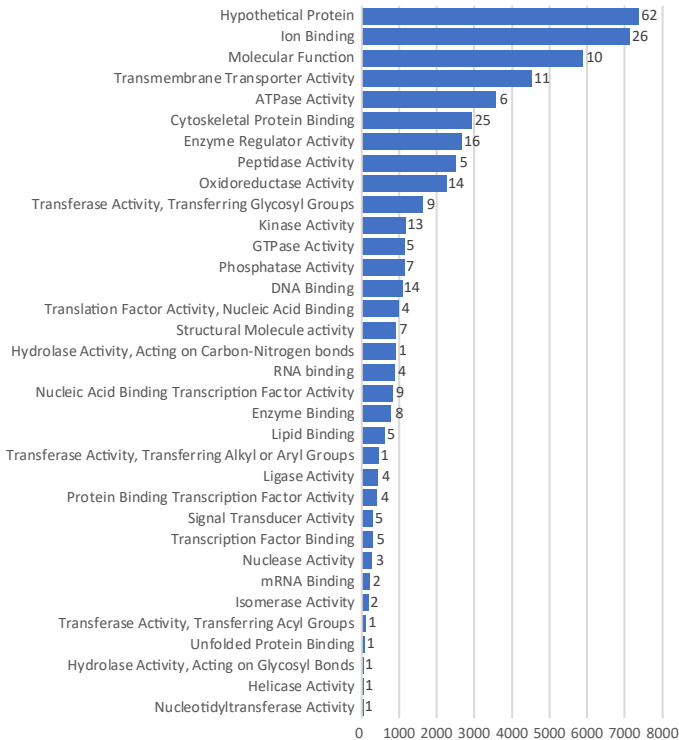

*Glossina* Combined RPKM Values  
Per Category

Supplement: Figure S3: Gene ontology analysis of bacteriocyte-enriched products associated with molecular functions [file rspb20170360supp3.pdf]

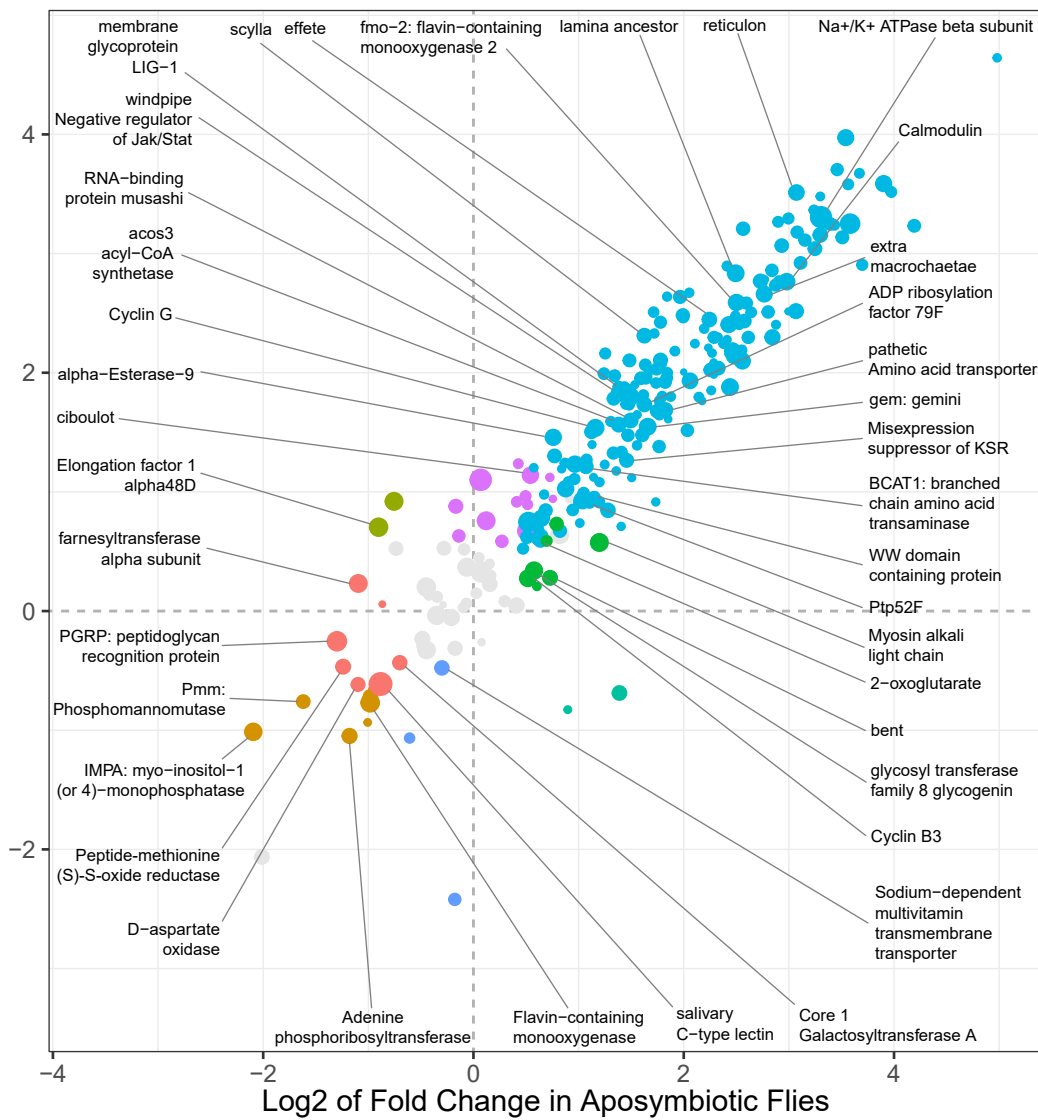

Supplement: Figure S4: Differential expression of bacteriocyte enriched genes in aposymbiotic and trypanosome infected flies with additional annotations [file rspb20170360supp4.pdf]

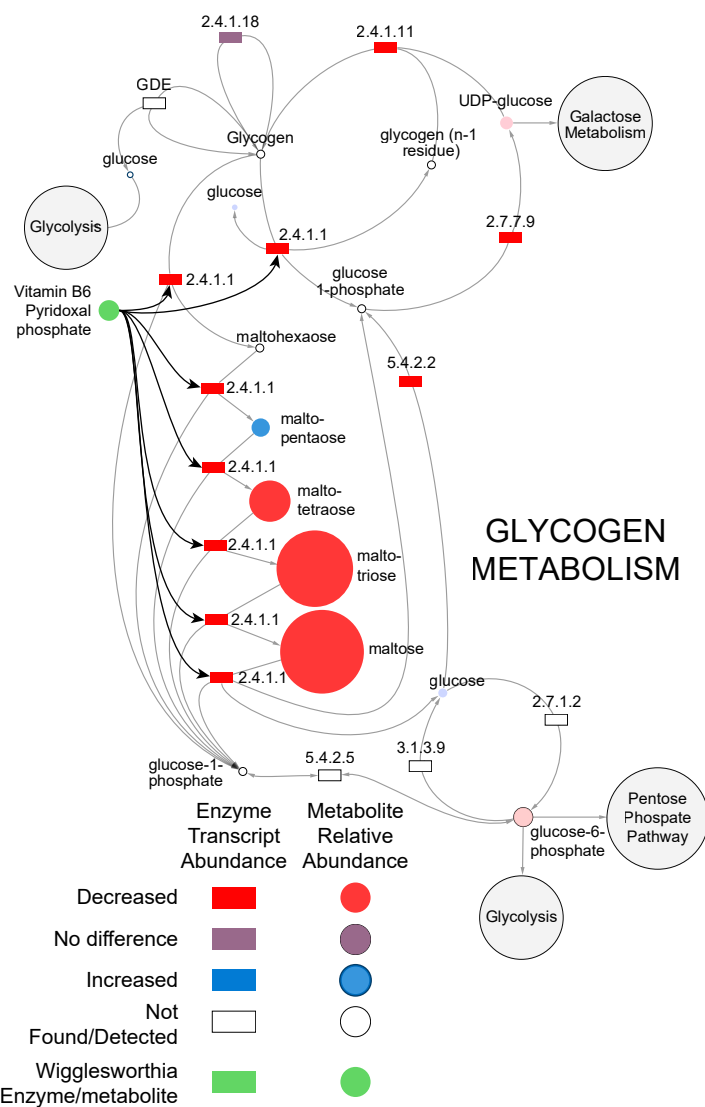

Supplement: Figure S5: Differential metabolite abundances and enzyme transcription between control and symbiont-cured tsetse in the glycogen metabolism pathway [file rspb20170360supp5.pdf]

# PURINE METABOLISM

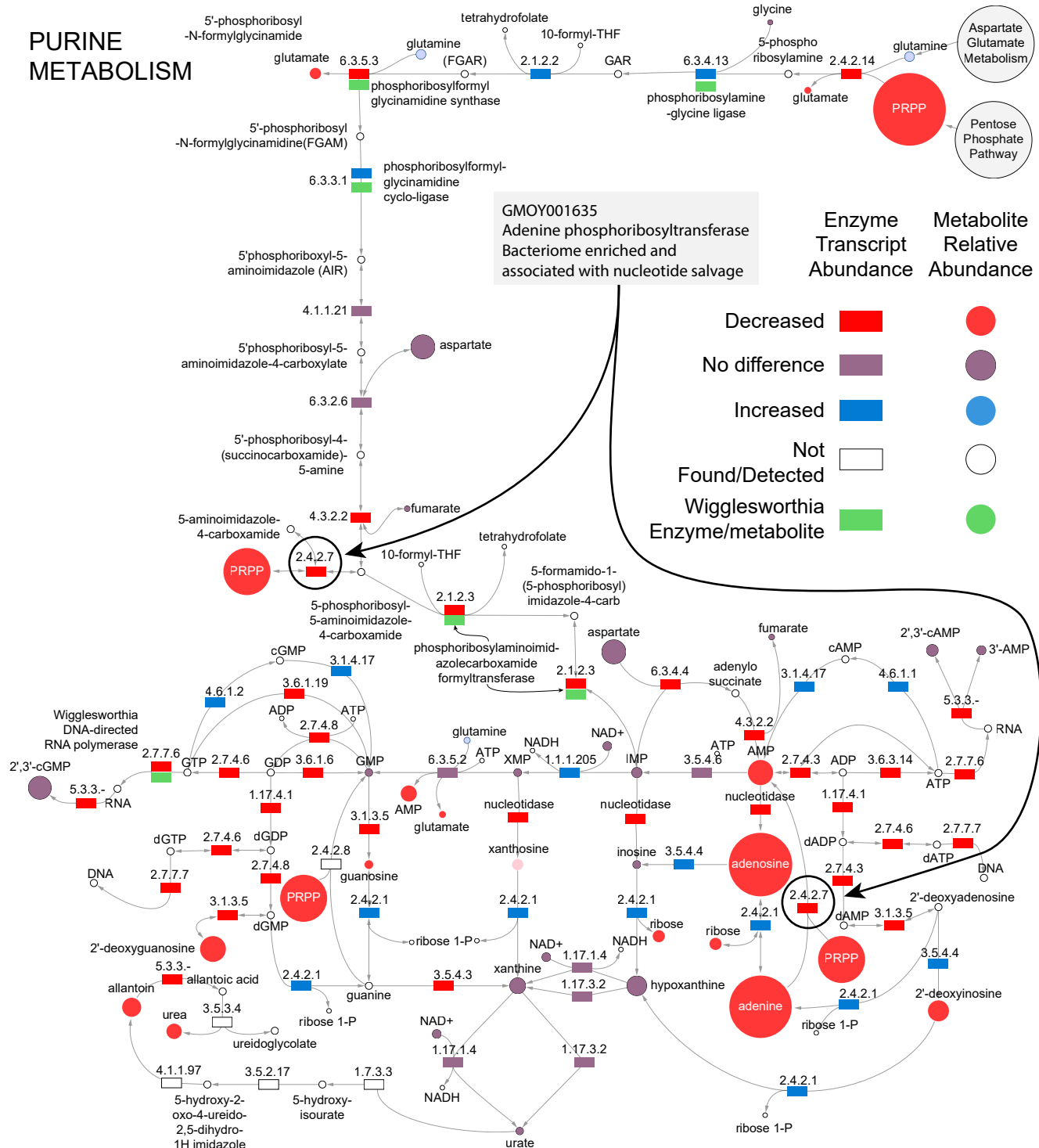

Supplement: Figure S6: Differential metabolite abundances and enzyme associated gene expression in the purine metabolism pathway between control and aposymbiotic tsetse [file rspb20170360supp6.pdf]
