# Supplementary material for: Unravelling the relationship between the tsetse fly and its obligate symbiont Wigglesworthia: transcriptomic and metabolomic landscapes reveal highly integrated physiological networks
Source: Proc Biol Sci. 2017 Jun 28;284(1857):20170360. doi: 10.1098/rspb.2017.0360 (PMC5489720; doi:10.1098/rspb.2017.0360)
Supplement: Figure S7: Differential metabolite abundances and enzyme associated gene expression in the pyrimidine metabolism pathway between control and aposymbiotic tsetse [file rspb20170360supp7.pdf]

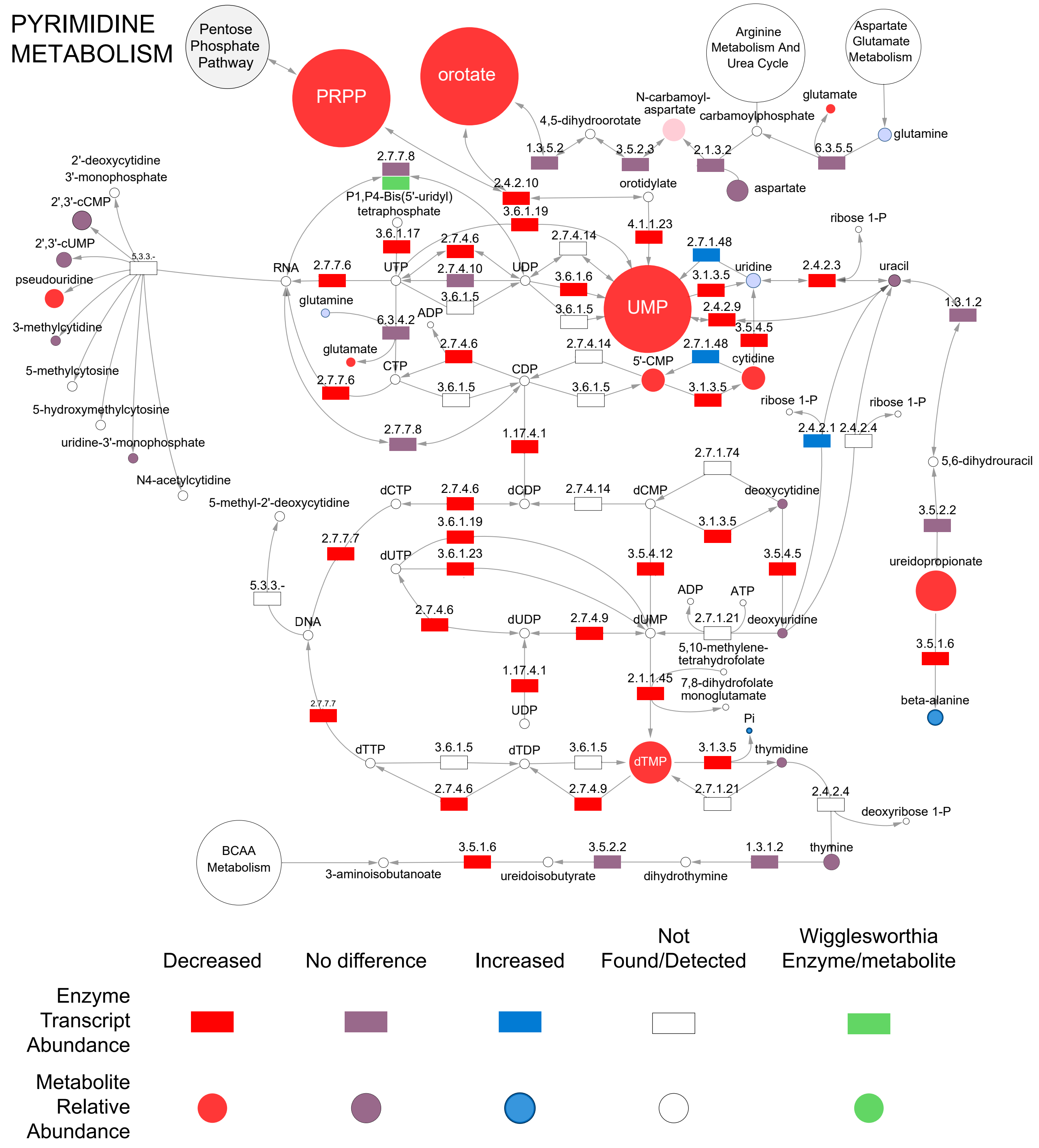
PYRIMIDINE METABOLISM
Pentose Phosphate Pathway
PRPP
orotate
Arginine Metabolism And Urea Cycle
Aspartate Glutamate Metabolism
N-carbamoyl-aspartate
4,5-dihydroorotate
carbamoylphosphate
glutamate
glutamine
1.3.5.2
3.5.2.3
2.1.3.2
6.3.5.5
aspartate
2.4.2.10
orotidylate
2.7.7.8
P1,P4-Bis(5'-uridyl) tetraphosphate
3.6.1.19
4.1.1.23
2'-deoxycytidine 3'-monophosphate
2',3'-cCMP
2',3'-cUMP
pseudouridine
3-methylcytidine
5-methylcytosine
5-hydroxymethylcytosine
uridine-3'-monophosphate
N4-acetylcytidine
5.3.3.-
RNA
2.7.7.6
UTP
3.6.1.17
2.7.4.6
2.7.4.10
UDP
2.7.4.14
3.6.1.6
3.6.1.5
UMP
2.7.1.48
3.1.3.5
uridine
2.4.2.3
uracil
ribose 1-P
2.4.2.9
1.3.1.2
3.5.4.5
glutamine
6.3.4.2
ADP
glutamate
CTP
CDP
5'-CMP
cytidine
2.4.2.1
2.4.2.4
1.17.4.1
5,6-dihydrouracil
2.7.1.74
dCTP
dCDP
dCMP
deoxycytidine
5-methyl-2'-deoxycytidine
3.5.2.2
ureidopropionate
2.7.7.7
dUTP
3.6.1.23
3.5.4.12
DNA
dUDP
2.7.4.9
dUMP
ATP
2.7.1.21
deoxyuridine
3.5.1.6
5,10-methylene-tetrahydrofolate
7,8-dihydrofolate monoglutamate
2.1.1.45
UDP
beta-alanine
Pi
dTTP
dTDP
dTMP
thymidine
deoxyribose 1-P
thymine
BCAA Metabolism
3-aminoisobutanoate
ureidoisobutyrate
dihydrothymine
Decreased
No difference
Increased
Not Found/Detected
Wigglesworthia Enzyme/metabolite
Enzyme Transcript Abundance
Metabolite Relative Abundance
