# Supplementary material for: Unravelling the relationship between the tsetse fly and its obligate symbiont Wigglesworthia: transcriptomic and metabolomic landscapes reveal highly integrated physiological networks
Source: Proc Biol Sci. 2017 Jun 28;284(1857):20170360. doi: 10.1098/rspb.2017.0360 (PMC5489720; doi:10.1098/rspb.2017.0360)
Supplement: Table S1: Transcriptomic and metabolomic datasets utilized in this study [file rspb20170360supp8.docx]

**Table 1: Transcriptomic and Metabolomic Datasets Utilized in this Study**

| **Dataset** | **Type** | **Biological**  **Replicates** | **Collection** | **SRA Numbers** |
| --- | --- | --- | --- | --- |
| Symbiotic Bacteriome | Transcriptome | 3 | Female flies 20 days post eclosion | Bioproject: PRJNA335358 |
| Control Symbiotic Midgut | Transcriptome | 3 | Female flies 17 days post eclosion | Bioproject: PRJNA368987 |
| Trypanosome Infected Midgut | Transcriptome | 3 | Female flies 17 days post eclosion | Bioproject: PRJNA368987 |
| Aposymbiotic Midgut | Transcriptome | 3 | Female flies 11 days post eclosion | Bioproject: PRJNA368970 |
| Symbiotic Bacteriome | Metabolome | 4 | Female flies 40 days post eclosion | Supplemental table S5 |
| Symbiont Cured Bacteriome | Metabolome | 4 | Female flies 40 days post eclosion | Supplemental table S5 |
| Symbiotic Hemolymph | Metabolome | 4 | Female flies 40 days post eclosion | Supplemental table S6 |
| Symbiont Cured Hemolymph | Metabolome | 4 | Female flies 40 days post eclosion | Supplemental table S6 |
